# Efficacy and safety of moxibustion in the treatment of cancer-related fatigue: A protocol for systematic review and meta-analysis

**DOI:** 10.1097/MD.0000000000024857

**Published:** 2021-03-05

**Authors:** Gen Deng, Xianbao Huang, Minfang Tu, Qinghong Cheng, Qi Qiu, Peiling Li, Zefeng Pan

**Affiliations:** a College of Acupuncture and Massage, Jiangxi University of Traditional Chinese Medicine; b Affiliated Hospital of Jiangxi University of Traditional Chinese Medicine, China.

**Keywords:** cancer-related fatigue, moxibustion, protocol, systematic review and meta-analysis

## Abstract

**Background:**

Cancer-related fatigue, a common symptom of cancer patients caused by the interaction of multiple factors, runs through the whole process of tumorigenesis, development, treatment, and prognosis. The main clinical manifestations are weakness, tiredness, exhaustion, fatigue, or slow movement, heavy limbs, low mood or irritability, sleep disturbance or lethargy, lack of attention, etc. CRF is different from the fatigue after daily body fatigue. It has no obvious relief or relief after rest or sleep, and exists for a long time in the relevant treatment and rehabilitation process. It seriously affects the physiological, psychological and social functions of patients, and reduces the quality of life of patients. Moxibustion therapy has shown strong advantages in the treatment of CRF, and the curative effect is accurate. Therefore, this paper will carry out a systematic evaluation and meta analysis of the efficacy and safety of moxibustion in the treatment of CRF.

**Methods:**

we will searching 8 electronic databases, including PubMed, Embase, Web of Science, Cochrane Library, the China National Knowledge Infrastructure, Chinese Science and Technology Periodical Database, Wanfang Database, and Chinese Biomedical Literature Database. We will search above electronic databases from the beginning to January 2021, without any language restriction. Clinical efficacy, including total effective rate or cure rate, clinical symptom integral, and recurrence rate will be accepted as the primary outcomes. The fatigue scale score, quality of life improvement rate will be used as secondary outcomes. RevMan 5.3 software will be used for statistical analysis. The result about the curative effect and safety of moxibustion for cancer-related fatigue will be presented as risk ratio for dichotomous data and mean differences with a 95% confidence interval for continuous data.

**Results:**

When this research program is completed, the relevant results can be obtained.

**Conclusions:**

The results of this study will provide reliable evidence for the efficacy and safety of moxibustion in the treatment of cancer-related fatigue.

**Ethics and dissemination:**

This article does not need to pass the ethics committee review, because this article does not involve the ethics question, only collates the related literature research.

**INPLASY Registration number:**

INPLASY202110072.

## 1 Introduction

Cancer-related fatigue (CRF), is a common symptom of cancer patients caused by the interaction of multiple factors, which runs through the whole process of tumor genesis, development, treatment, and prognosis.^[1]^ CRF is different from the fatigue after daily body fatigue. It has no obvious relief or relief after rest or sleep, and exists for a long time in the relevant treatment and rehabilitation process. It seriously affects the physiological, psychological and social functions of patients, and reduces the quality of life of patients.^[2]^ Although a large number of studies have been conducted on cancer induced fatigue at home and abroad, the pathogenesis of cancer induced fatigue is still unclear at present. The latest guidelines define CRF as a painful, persistent, subjective feeling of physical, emotional, or cognitive fatigue that is not consistent with recent activity, is related to cancer or cancer treatment, and interferes with daily life.^[3]^ Clinically, CRF is usually treated with western medicine, but the efficacy is short, the recurrence rate is high, the toxic and side effects of drugs are large, and even the adverse reactions of these drugs aggravate the degree of cancer-related fatigue of patients instead.^[4]^ CRF is not only a hot topic in current research, but also an urgent problem in improving the quality of life and prolonging the survival time of cancer patients in clinical practice.

Moxibustion is a kind of traditional Chinese medicine external therapy, which has the characteristics of simple, safe, effective, and nontoxic side effects.^[5,6]^ In recent years, there have been more and more clinical reports on moxibustion therapy for CRF.^[7–9]^ However, there is still a lack of systematic evaluation on the efficacy and safety of moxibustion therapy for CRF in clinical practice. Therefore, the effectiveness and safety of moxibustion in the treatment of CRF will be systematically evaluated and meta-analyzed in this paper.

## 2 Methods

### 2.1 Study registration

This protocol was registered with the International Platform of Registered Systematic Review and Meta-Analysis Protocols (INPLASY) on January 19, 2021 and was last updated on January 19, 2021 (registration number INPLASY202110072).

### 2.2 Inclusion criteria for study selection

#### 2.2.1 Types of studies

Clinical randomized controlled trials (RCTs) containing moxibustion for CRF will be included, with no limitation of language and publication status.

#### 2.2.2 Types of participants

There are clear and recognized diagnostic criteria and efficacy criteria, and all patients are diagnosed as CRF, regardless of gender, age, and origin of the case.

#### 2.2.3 Types of interventions

##### 2.2.3.1 Experimental interventions

Moxibustion therapy will include all therapies using any type of moxibustion, such as indirect moxibustion, direct moxibustion, heat-sensitive moxibustion, and so on. Mixed therapies based on moxibustion will also be included. Moxibustion therapy, or mixed therapies based on moxibustion will also be include.

##### 2.2.3.2 Control interventions

The control group will receive one of the following treatment methods: conventional pharmacological therapy, no treatment, and placebo.

#### 2.2.4 Types of outcome measures

##### 2.2.4.1 Primary outcome

Clinical efficacy, including total effective rate or cure rate, clinical symptom integral, and recurrence rate will be accepted as the primary outcomes.

##### 2.2.4.2 Secondary outcomes

The fatigue scale score, quality of life improvement rate will be used as secondary outcomes.

### 2.3 Exclusion criteria

Nonrandomized controlled trials; no exact diagnostic scale or therapeutic scale; no moxibustion as the main treatment in the experimental group, and moxibustion therapy was found in the control group. Repeated literature; theory and review literature; animal experiments; nursing research.

### 2.4 The retrieval methods and strategies of this study

#### 2.4.1 Electronic database retrieval

We will search 8 electronic databases, including PubMed, Embase, Web of Science, Cochrane Library, the China National Knowledge Infrastructure, Chinese Science and Technology Periodical Database, Wanfang Database, and Chinese Biomedical Literature Database. We will search above electronic databases from the beginning to January 2021, without any language restriction. And will searching the relevant literature by combining subject words with free words, search terms consist of disease (“cancer-related fatigue” or “CRF” or “cancer fatigue” or “Empty labour”) and intervention (“moxibustion” or “moxa” or “moxabustion” or “cauterize” or “moxa stick moxibustion”) and research types (“randomized controlled trial” or “controlled clinical trial” or “random trials” or “RCT”). The PubMed search strategy is shown in Table 1.

**Table 1:** Retrieval strategies in PubMed.

ID	Query
#1	“Moxibustion”[Mesh]
#2	(((moxibustion[Ti/Ab]) OR (moxabustion[Ti/Ab])) OR (cauterize[Ti/Ab])) OR (moxa stick moxibustion[Ti/Ab])
#3	#1 OR #2
#4	“cancer-related fatigue”[Mesh]
#5	(((cancer fatigue[Ti/Ab]) OR (cancer related fatigue[Ti/Ab])) OR (CRF[Ti/Ab])) OR (Empty labour[Ti/Ab])
#6	#4 OR #5
#7	(((randomized controlled trial[Ti/Ab]) OR (random trials[Ti/Ab])) OR (controlled clinical trial[Ti/Ab])) OR (RCT[Ti/Ab])
#8	#3 AND #6 AND #7

#### 2.4.2 Searching other resources

We will combine manual retrieval of literature resource database to search relevant conference papers that meet the inclusion criteria. In addition, the grey literature, as well as ongoing and recently completed studies, will be searched on Clinicaltrials.gov.

### 2.5 Data extraction and management

#### 2.5.1 Literature inclusion and data extraction

**Figure 1:**
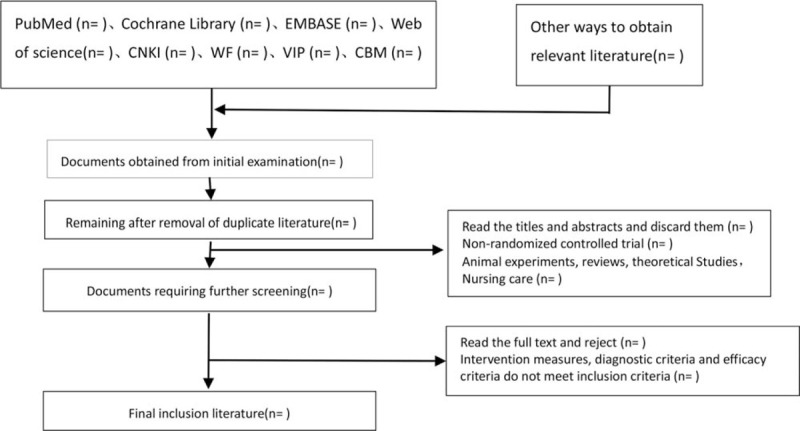
Flow chart of literature incorporation.

The 2 researchers independently read the title and abstract of the literature we obtained, read the full text of the trials that might meet the inclusion criteria to determine whether the inclusion criteria were truly met, and discussed the conflicting literatures or let the third researcher decide whether to include them. Two researchers independently extracted data from the included studies, including study design, intervention measures and methods, measurement indicators, results, methodological contents such as hidden grouping and blind method, etc., and a third evaluator checked the consistency of the data. If the required information is incomplete, we will contact the original author for the required data. The inclusion process of this study will be carried out as shown in Figure 1.

#### 2.5.2 Methodological quality evaluation

Two evaluators independently select the literature according to the inclusion and exclusion criteria and cross-check. In case of disagreement, a third evaluator will assist in the decision. The extracted data included the first author, year of publication, number of patients, age, gender, intervention measures, outcome indicators, etc. The Jadad scale to evaluate quality into literature, including: random sequence (right 2 points, 1 points not clear, inappropriate 0), distribution, hidden (right 2 points, 1 points not clear, inappropriate 0), blinded (right 2 points, 1 points not clear, inappropriate 0), lost to follow-up and exit (describe 1 points, not describe 0); 0–3 is classified as low quality and 4–7 as high quality.

### 2.6 Statistical analysis

#### 2.6.1 Quantitative data synthesis

Meta analysis will be performed using Rev Man 5.3.0 software. The odds ratio (OR) and its 95% confidence interval (CI) will be used as the counting data, while the weighted mean difference (WMD) and its 95% CI will be used as the measurement data.

#### 2.6.2 Assessment of heterogeneity

The heterogeneity test will be carried out first among all studies, *I*^2^ test will be used. When *P* > .1 and *I*^2^ < 50%, the fixed effect model will be used; otherwise, the random effect model will be used. When the clinical heterogeneity between the 2 studies is large, only descriptive analysis will be performed.

#### 2.6.3 Publication bias

When the number of qualified RCTS is sufficient, we will use the inverted funnel Egger to test the potential publication bias.

#### 2.6.4 Subgroup analysis

If the necessary data are available, subgroup analysis will be carried out according to different factors based on the type of CRF, treatment cycle, each moxibustion treatment time, and the type of intervention in the control group.

#### 2.6.5 Sensitivity analysis

The purpose of sensitivity analysis is to determine the sources and confounding factors of heterogeneity. If the trial data is sufficient, low or high quality studies will be excluded one by one for sensitivity analysis.

## 3 Discussion

Cancer-related fatigue, is a common symptom of tumor patients caused by multi-factor interaction, which always runs through the whole process of tumorigenesis, development, treatment, and prognosis.^[1]^ The main clinical manifestations are weakness, fatigue, exhaustion, fatigue, or slow movement, heavy limbs, depression or irritability, sleep disorders or drowsiness, attention deficit.^[10]^ CRF is different from the fatigue after daily physical fatigue, after rest, sleep after no significant relief or relief, and in the related treatment and rehabilitation process for a long time, seriously affect the physical, psychological and social functions of patients, reduce the life of patients Quality.^[2]^ According to the results of the investigation, the incidence of fatigue in patients with malignant tumors treated can reach 70% to 100%,^[11]^ of which about 65% of patients with malignant tumors receiving radiotherapy will develop fatigue, while about 82% to 96% of patients with malignant tumors who have received chemotherapy will develop fatigue. About 70% of patients receiving biotherapy will develop fatigue.^[12]^ Although a large number of studies have been carried out on cancer-related fatigue at home and abroad, the pathogenesis of cancer-related fatigue is not clear at present. However CRF, the curative effect is short, the recurrence rate is high, the side effects of drugs are great, and even the adverse reactions of these drugs are counterproductive And aggravated the degree of cancer-related fatigue in patients.^[4]^ CRF is a hot issue in current research, and it is also a difficult problem to improve the quality of life and prolong the life time of cancer patients.

Moxibustion is an external treatment of traditional Chinese medicine, which has the functions of warming the meridian and dispersing cold, tonifying deficiency, and strengthening the qi. The therapy avoids the patients’ fear of acupuncture and acupuncture, and has obvious clinical application advantages. Moxibustion can play the role of warming meridian and clearing collaterals, activating blood circulation and removing blood stasis, warming yang and tonifying deficiency, regulating viscera and balancing yin and yang, and enhancing the ability of human body to resist evil, which belongs to the category of strengthening and resisting evil. Clinical research on moxibustion treatment CRF has been increasing in recent years. Modern research shows that the therapeutic effect of moxibustion is combined with the physical and chemical action of moxibustion with the special action of acupoints and the special way of meridian and produce a kind of “comprehensive effect.”^[13]^ Moxibustion stimulates the body's own endogenous regulatory system through multiple targets and pathways through the comprehensive stimulation of the body's warm heat stimulation, light radiation, and moxibustion production (smoke). It promotes the production of endogenous protective substances, stimulates the related acupoints, and stimulates the meridian conduction in the whole body to achieve the purpose of treating diseases.^[14–16]^ At the same time, the study also shows that moxibustion has the functions of bidirectional regulation of body immunity, anti-infection, antitumor, analgesia and promoting metabolism, delaying aging, etc. In the treatment of malignant tumors, it can inhibit tumor growth, alleviate adverse reactions of radiotherapy and chemotherapy, and assist radiotherapy, chemotherapy and surgical treatment, to deal with some complications, prolong the survival time and improve the quality of life.^[14–17]^ Therefore, it is necessary to systematically evaluate the treatment of CRF by moxibustion in this study, which can provide evidence-based medicine evidence for future clinical guidance of the treatment of CRF by moxibustion.

## Author contributions

**Data curation:** Gen Deng, Zefeng Pan.

**Formal analysis:** Gen Deng, Qinghong Cheng.

**Investigation:** Gen Deng, Peiling Li.

**Methodology:** Minfang Tu, Peiling Li.

**Project administration:** Xianbao Huang, Qinghong Cheng.

**Resources:** Qinghong Cheng.

**Software:** Minfang Tu, Zefeng Pan.

**Supervision:** Xianbao Huang, Qi Qiu.

**Validation:** Minfang Tu.

**Visualization:** Minfang Tu, Qi Qiu.

**Writing – original draft:** Gen Deng, Xianbao Huang.

**Writing – review & editing:** Gen Deng, Xianbao Huang.
